# Associations between circulating obesity-related biomarkers and prognosis in female breast cancer survivors: a systematic review of observational data in women enrolled in lifestyle intervention trials

**DOI:** 10.1186/s12885-022-10274-3

**Published:** 2022-11-18

**Authors:** Dorothy Meyer, Belén Pastor-Villaescusa, Sophie Michel, Hans Hauner, Dagmar Hauner

**Affiliations:** Institute of Nutritional Medicine, Else Kröner-Fresenius-Centre for Nutritional Medicine, School of Medicine, Technical University of Munich, Georg-Brauchle-Ring 62, 80992 Munich, Germany

**Keywords:** Systematic review, Breast cancer recurrence, Disease-free survival, Breast cancer mortality, Biomarker, Obesity

## Abstract

Obesity plays an important role in the development and progression of breast cancer via various oncogenic pathways. However, the biological mechanisms underlying this relationship are not fully understood. Moreover, it is unclear whether obesity-related and further associated biomarkers could be suitable targets for lifestyle interventions. This systematic review was conducted to examine relationships between obesity-related blood parameters and prognosis for breast cancer survivors enrolled in lifestyle intervention studies. A systematic, computerized literature search was conducted from inception through August 26th, 2020 in PubMed, EMBASE, and CENTRAL. The focus was on observational data from randomized controlled lifestyle intervention trials investigating associations between selected baseline biomarkers, measured in remission, and breast cancer recurrence, breast cancer mortality and/or all-cause mortality. Four studies with data from 5234 women met the inclusion criteria.

Studies herein provide moderate evidence that bioavailable or serum testosterone may be positively linked to breast cancer recurrence and inversely linked to disease-free survival. Limited evidence suggests no associations with circulating estradiol or insulin levels on prognosis outcomes, whereas HDL cholesterol was inversely associated with breast cancer recurrence. For some other biomarkers, such as growth factors, adipokines, and CRP, the evidence for associations with disease prognosis was too weak to draw conclusions.

Overall, despite potential candidates, there is insufficient evidence to confirm or refute that obesity-related biomarkers and sex hormones have a prognostic value for breast cancer survival. More longitudinal studies in breast cancer survivors to examine the clinical utility of obesity-related biomarkers are needed.

**Supplementary Information:**

The online version contains supplementary material available at 10.1186/s12885-022-10274-3.

## Background

Overweight and obesity play a critical role in both the development and prognosis of breast cancer [1–3]. Evidence suggests that higher body weight negatively affects survival in both pre- and post-menopausal women [2–6]. Our current understanding of the biological mechanisms underlying the relationship between obesity and breast cancer survival is incomplete. Low-grade chronic inflammation is a hallmark of overweight and obesity [7], and investigating the role of inflammation in cancer initiation and progression has gained considerable interest [8]. Several other factors and mechanisms triggered by obesity may be implicated in tumor development and progression, such as modulations in sex steroids, insulin and insulin resistance, altered secretion of adipokines [9], and the activation of insulin-like growth factor (IGF) pathways [10].

Research has demonstrated that lifestyle interventions focusing on a healthy diet and increased physical activity can lead to weight loss [11] and improvements in prognosis outcomes for breast cancer survivors [12]. The benefits of intentional weight loss may partly result from reduced concentrations of circulating factors associated with obesity and disease progression, such as sex hormones and inflammatory markers [13]. However, few studies have explored whether changes in concentrations of obesity-related biomarkers influence cancer progression or overall prognosis [14, 15]. Prognostic, or risk prediction biomarkers, predict the development of disease and can be a tool for making treatment decisions [16]. Thus, the goal of this systematic review was to examine relationships between obesity-related blood parameters and prognosis in breast cancer survivors enrolled in lifestyle intervention studies. We were interested in biomarkers measured at baseline, i.e. before the start of the intervention, in women determined to have no evidence of disease at recruitment.

## Main text

### Materials and methods

This review was conducted according to the Preferred Reporting Items for Systematic Reviews and Meta-Analyses guidelines (PRISMA 2020) [17] (Additional file 1). The trial protocol was registered in the International Prospective Register of Systematic Reviews (PROSPERO) under the registration number CRD42020203013. This systematic review is embedded in a larger research project on biomarkers and breast cancer survival.

#### Inclusion and exclusion criteria

Study inclusion and exclusion criteria were defined using the PICOS/PECOS framework (participants, intervention/exposure, control, outcome, study design) [18] (Table 1). The review considered randomized controlled trials (RCTs) investigating the effect of lifestyle interventions in female breast cancer survivors that also analyzed observational data on associations between baseline biomarkers and prognosis. Lifestyle interventions were considered to include diet, physical activity, or a combination of both. RCTs with breast cancer survivors undergoing surgical-, pharmacological-, or dietary supplementation interventions at the time of recruitment were not considered. Studies were considered if the women had completed surgery and chemotherapy for breast cancer and were determined to be disease-free before recruitment. Women receiving ongoing adjuvant treatments, such as hormonal or immunological therapies were not excluded. Studies restricted to women with specific medical conditions (e.g., lymphedema) were excluded. In situ and metastatic breast cancer cases were not considered, nor were cancers caused by specific gene mutations (e.g., BRCA1 or BRCA2).

**Table 1:** Inclusion and exclusion criteria

Inclusion criteria	Exclusion criteria
• Women ≥18 years	• In situ (stage 0) and metastatic (stage IV)
• Breast cancer survivors who had completed surgery and chemotherapy at the time of recruitment	• Breast cancer caused by gene mutations (e.g. BRCA1 or BRCA2)
• All BMI categories	• Studies restricted to participants with specific conditions (such as fatigue, lymphedema, or bone loss)
	• Studies with surgical, pharmacological, or dietary supplementation interventions

The search considered obesity-related circulating biomarkers (e.g. related to glucose metabolism and insulin resistance, lipid metabolism, inflammation, or secreted products from adipose tissue) and other circulating biomarkers, including metabolites and metabolite signatures from metabolomics studies. No criteria related to study sample size were employed. References in English, Spanish or German were considered. Conference abstracts, case reports, ecological studies, and letters to the editor were excluded.

#### Search strategy

We conducted a systematic literature search in the electronic databases PubMed, EMBASE, and CENTRAL from inception to 26th August, 2020. The search strategy combined controlled vocabulary / index terms and free-text / keywords related to: “breast cancer”, “cancer survivor”, “biomarker”, “prognosis”, “obesity” and “lifestyle intervention”. No built-in filters were applied to ensure the sensitivity of the search. Full search strategies used for the respective databases are presented in Additional file 2. Further, reference lists of included studies and relevant systematic reviews were hand-searched independently by two reviewers (BPV and SM) for additional studies. Finally, complementary internet searches in Google and Google Scholar were conducted and updated until November 2020 to identify further references. While the search strategy was developed for a larger research project that extends beyond the scope of the current review, this does not affect the sensitivity of the search strategy with respect to the current research question.

#### Study selection, data extraction and quality assessment

EndNote software X8 (Thomson Reuters, New York City, NY) was used to export the identified references. After removing duplicates, two researchers (BPV and SM) independently screened the titles, abstracts, and full-texts according to the aforementioned selection criteria. Discrepancies were resolved by discussion with other researchers (DH and HH). During the full-text screening, a list with references not meeting eligibility was kept, along with notes on reasons for exclusion. Additional file 3 provides a list of excluded references with reasons for exclusion.

Data on the study design, population characteristics, exposures, comparator/control groups, outcomes, statistical methods, and results were extracted independently by two researchers (BPV and DM) and reviewed by DH. For each outcome, hazard ratios, odds ratios, confidence intervals and *p* values were extracted, as well as means ± standard deviations, when applicable. Study protocols were additionally considered if available. Differences were resolved by discussion among BPV, DM, DH and HH. Authors of eligible studies were contacted via email to obtain missing data.

Two researchers (BPV and DM) independently assessed the included studies for risk of bias using the Newcastle-Ottawa scale (NOS) [19]. Any discrepancies were resolved by discussion, and, if necessary, after consulting other researchers (DH and HH). The NOS assigns points, or “stars” for high-quality characteristics in each of the three domains: 1) selection of participants and study design (max. Four points), 2) comparability of groups (max. Two points), and 3) ascertainment of exposure and outcomes (max. Three points). Scores for overall study quality were assigned as follows: low quality (0–3 points), moderate quality (4–6 points), and high quality (7–9 points).

## Results

**Fig. 1:**
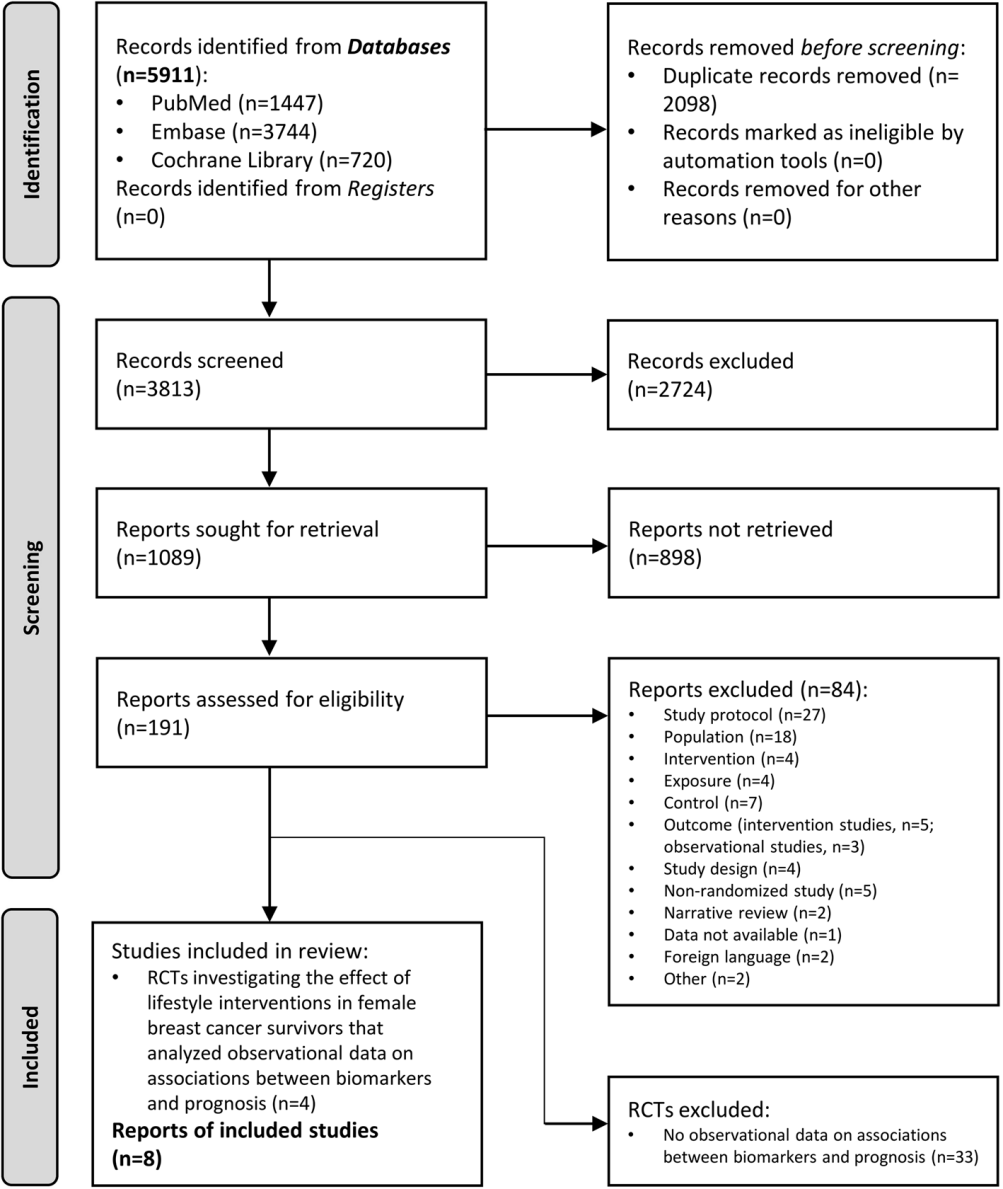
PRISMA flow chart

The screening process for eligible studies is described in Fig. 1. Searching electronic databases yielded 5911 records. After removing duplicates and excluding any records that did not meet the inclusion criteria, a total of 191 studies were assessed for eligibility. Complementary internet searches yielded no additional studies. According to PICOS/PECOS criteria, 84 studies were excluded. Four RCTs were found to report data on secondary analyses/nested observational studies relating to associations between clinical biomarkers and prognosis (defined as either breast cancer recurrence, disease-free survival, breast cancer mortality or all-cause mortality). The review process ultimately identified eight reports from the four RCTs that met the established inclusion criteria.

PRISMA flow chart detailing database searches, abstracts screened, and full texts retrieved and included in the systematic review.

The results of the NOS quality assessment are presented in Table 2. Since the WHEL study reported cohort and case-control data, the risk of bias evaluation was carried out for both types of study designs. Three studies received high-quality scores and one a moderate-quality score.

**Table 2:** Newcastle-Ottawa Scale for Non-Randomized Clinical Trials

Study	Selection (max 4•)	Comparability (max 2•)	Outcome (cohorts)/ Exposure (case-control) (max 3•)	Total score
DIANA-2	•••	•	•••	7
DIANA-5	••••	••	••	8
PACThe study	••	–	•••	5
WHEL study				
*Cohort*	••••	••	•••	9
*Case-control*	••••	••	•••	9

### Study characteristics

A total of 5234 women were included in the four studies. All women were reported to have no clinical evidence of disease at the time of recruitment. Biomarkers of interest were reported in Berrino et al. [20], Pasanisi et al. [21] and Pasanisi et al. [22] from the DIANA 2 study. Several biomarkers were investigated in the DIANA-5 study by Berrino and colleagues [23]. Vasson and colleagues [24] reported baseline biomarker data from the PACThe study. The WHEL study reported on circulating biomarkers in Emond et al. [25], Al-Delaimy et al. [26] and Villaseñor et al. [27]. A complete list of studies with their main characteristics and investigated biomarkers is provided in Table 3. DIANA-2 [20–22] and WHEL [25–27] consisted of healthy diet interventions, while DIANA-5 [23], and PACThe [24] included both diet and exercise programs. Biomarkers in these studies were measured before lifestyle interventions were implemented. DIANA-2 [20–22], DIANA-5 [23] and PACThe [24] were conducted in Europe, while WHEL [25–27] took place in the United States. Sample sizes ranged from 107 to 2919 women. Most of the studies predominantly included postmenopausal women (60–100%) except DIANA-5, with 55% of women reported to be premenopausal [23].

**Table 3:** Characteristics of included studies reporting associations between blood biomarkers and prognosis

Authors (year) or study name, country	Sample characteristics	Exposures ^**a**^ (Measurement technique)	Statistical method, adjustment factors	Outcomes	Main significant findings
**DIANA-2, Italy**					
Berrino et al. (2005) [20]	107 postmenopausal breast cancer survivors. Mean age: 56.8 years. Patients with normal-, or overweight/obesity. Operated for breast cancer at least a year prior to enrolment. HT: about 38% of women were under tamoxifen treatment during the RCT. Subtypes: ER-, ER+, PR-, PR+, unknown. Stage not reported. Serum samples collected at baseline of the RCT. Follow-up: 5.5 years	Serum testosterone, estradiol, SHBG, glucose, insulin (Radioimmunoassay)	Cox proportional hazards models, adjusted for HR T (size of the primary) and N (axillary node status) with/without tertiles of baseline testosterone ^b^	Recurrence (local relapse, distant metastasis, contralateral breast cancer)	Recurrent patients: ↑ testosterone, estradiol, glucose vs. patients without recurrence (0.52 vs. 0.38 ng/ml, *p* < 0.001; 8.06 vs. 5.52 pg/ml, *p* = 0.02; 96 vs. 91 mg/dl, p = 0.02, respectively). In tertiles: only ↑ testosterone (3rd tertile) was significantly associated with ↑ RR (HR: 7.19, 95% CI 2.42–21.35, p not shown). No association with RR were observed for estradiol, SHBG, fasting glucose and insulin
Pasanisi et al. (2006) [21]	110 postmenopausal breast cancer survivors. Mean age: 56.8 years. Patients with normal-, or overweight/obesity, 16 women with MetS. Operated for breast cancer at least a year prior to enrolment. HT: about 38% of women were under tamoxifen treatment during the RCT. Subtypes: ER-, ER+, PR-, PR+. Stage not reported. Serum samples collected at baseline of the RCT. Follow-up: 5.5 years	Serum testosterone (Radioimmunoassay), glucose, insulin, triglycerides, HDL-c	Cox proportional hazards models, adjusted for age with/without pathological prognostic factors ^b^	Recurrence (local relapse, distant metastasis, contralateral breast cancer)	Women with MetS and testosterone > 0.40 ng/mL: ↑ RR vs. women without MetS and testosterone ≤0.40 ng/mL (HR: 6.7, 95% CI 2.3–19.8, p not shown). No significant associations between each component of MetS and RR
Pasanisi et al. (2008) [22]	110 postmenopausal breast cancer survivors. Mean age: 56.8 years. Patients with normal-, or overweight/obesity. Operated for breast cancer at least a year prior to enrolment. HT: about 38% of women were under tamoxifen treatment during the RCT. Subtypes: ER-, ER+, PR-, PR+. Stage not reported. Serum samples collected at baseline of the RCT. Follow-up: 5.5 years	IGF-1, PDGF (radioimmunoassay), fructosamine, CRP (Hitachi modular automatic analyzer)	Cox proportional hazards models, adjusted for pathologic prognostic factors with (FAM)/without tertiles of body weight and serum testosterone ^b^	Recurrence (local relapse, distant metastasis, contralateral breast cancer)	Recurrent patients: ↑ PDGF vs. patients without recurrence (11.9 ng/mL vs. 9.4 ng/mL, respectively, p = 0.01). In FAM, PDGF and IGF-1 combined, women with ↑ PDGF and ↑ IGF-1 (> their median value) had ↑ RR vs. women with ↓ PDGF and ↓ IGF-1 (HR: 6.4, 95% CI, 1.5–26.7, p not shown). In quartiles: no biomarkers showed significant associations with RR
**DIANA-5, Italy**					
Berrino et al. (2014) [23]	2092 breast cancer survivors (about 45% postmenopausal) at high RR because of metabolic or endocrine milieu. Mean age: 51.4 years. 897 women with WC: ≥ 85 cm, 419 women with MetS. Operated for breast cancer on average 1.7 years (0–5 years) before enrolment. HT: not reported. Subtypes: ER+, PR+, HER2+. Stage: I-III. Plasma samples collected at baseline. Follow-up: median of 2.8 years	Glucose, HOMA-IR, triglycerides, HDL-c (Routine laboratory techniques)	Logistic regression models, adjusted for age, education, stage at diagnosis, ER expression	Recurrence (loco-regional recurrences, distant metastasis and new primary breast cancer)	No significant associations between fasting glucose, HOMA-IR and recurrence. ↓ HDL-c (OR 1.83, 95% CI 1.24–2.70, p not shown) and ↑ TG (OR 1.58, 95% CI 1.01–2.46, p not shown) were associated with ↑ RR
**PACThe study, France**					
Vasson et al. (2020) [24]	113 breast cancer survivors (about 60% postmenopausal). Mean age: 52 years. Mean BMI: 27.9 kg/m^2^, Patients with under-, normal-, overweight/obesity. Operated for breast cancer and completed chemotherapy and/or radiotherapy < 9 months before the randomization. HT: most women were under tamoxifen treatment during the RCT. Subtypes: HoR+, HER2+. Stage: not reported. Plasma samples collected at baseline of the RCT. Follow-up: 7 years	HDL-c (colorimetry methods), plasma testosterone (ELISA), CA 15–3 (Clermont Ferrand) Categorized in quartiles	Survival curves using Kaplan-Meier’s method, comparison of curves using the Log-rank test. Cox proportional hazard model, no adjustments	Disease-free survival (absence of local or distant (nodes, metastasis, and/or contralateral breast cancer))	In quartiles: ↑ HDL-c was associated with the best survival without recurrence (p = 0.047). ↓ testosterone and CA 15–3 were associated with longer disease-free survival (p = 0.001 and 0.03, respectively). Based on survival curves, testosterone was relevant for disease-free survival only in patients treated with HT (p = 0.012 vs. p = 0.69 for patients with and without HT). With the Cox model, only ↑ testosterone was associated with ↑ RR (HR 5.06, 95% CI 1.66–15.41, p = 0.004)
**WHEL study, USA**					
Emond et al. (2011) [25]	447 postmenopausal breast cancer survivors without baseline hot flash symptoms. Age: 18–70 years. Metabolic diseases and BMI not reported. Operated for breast cancer and had completed chemo- and/or radiotherapy. HT: about 63% of women were under tamoxifen treatment at baseline of the RCT. Subtypes: not reported. Stage: I II, III. Serum samples collected at baseline of the RCT. Follow-up: mean of 7.3 years	Bioavailable and total estradiol, bioavailable and total testosterone (radioimmunoassay), SHBG (two-site chemiluminometric sandwich assay)	Cox proportional hazards models, adjusted for intervention arm, baseline hormone concentration, site, antiestrogen use, # of positive nodes, tumor size, oophorectomy status, and previous hormone replacement therapy use ^b^	Recurrence (local, regional, or distant, or new primary events)	↑ Bioavailable testosterone associated with ↑ RR (HR for one unit increase in ln-transformed values: 1.69, 95% CI 1.00–2.84, p = 0.049). No significant associations with estradiol or SHBG were observed
Al-Delaimy et al. (2011) [26]	510 Recurrent breast cancer patients (about 75% postmenopausal) vs. 510 non-recurrent breast cancer patients (80% postmenopausal). Age: 18–70 years. Metabolic diseases and BMI not reported. Operated for breast cancer and had completed chemo- and/or radiotherapy. HT: about 58% of women were under tamoxifen treatment at baseline of the RCT. Subtypes: ER-, ER+. Stage: I II, III. Serum samples collected within 6 months after the enrollment. Follow-up: mean of 7.3 years	Insulin, leptin (Luminex technology), IGF-1, IGFBP-1, IGFBP-3, adiponectin (immunoassay) ^c^	Cox proportional hazards models, adjusted for tamoxifen use and menopausal status ^b^	Breast cancer event (recurrent/new primary breast cancer event)	Insulin, IGFBP-1, IGFBP-3, leptin, and adiponectin did not predict a breast cancer event in the 188 case-control pairs. No significant associations were found in the larger sample of 510 case-control pairs analyzed for IGF-1 (in quartiles and as a continuous measurement)
			Matched on randomization		
Villaseñor et al. (2013) [27]	2919 breast cancer survivors (about 80% postmenopausal). Age: 27–74 years. Patients with under-, normal-, or overweight/obesity. Operated for breast cancer and had completed chemo- and/or radiotherapy HT: about 69% of women were under tamoxifen treatment at baseline of the RCT. Subtypes: ER+/PR+, ER+/PR-, ER−/PR+, ER−/PR-, HER2+, HER2-, unknown. Stage: I II, IIIA. Serum samples collected at a mean of 23.6 months post-diagnosis. Follow-up: mean of 7.4 years	hsCRP (High-sensitivity electrochemiluminescence assay)	Cox proportional hazards models, adjusted for age at diagnosis, time since diagnosis, race/ethnicity, and stage and grade (model 1), model 1 plus BMI (model 2), model 2 plus anti-estrogen medication use and ER/PR status (model 3) ^b^	Additional breast cancer events (recurrence or new primary breast cancer) and breast cancer mortality	↑ InCRP was associated with ↑ additional breast cancer events (HR 1.13, 95% CI 1.03–1.24, p = 0.03, model 3) and ↑ breast cancer mortality (HR 1.16 1.01–1.31, p t = 0.03, model 3). In cut-off: ↑ hsCRP levels (> = 10 mg/L) vs. no inflammation levels (< 1,0 mg/L) were associated with ↑ additional breast cancer events (HR 1.65, 95% CI 1.15–2.38, *p* = 0.03, model 3), and ↑ breast cancer mortality (HR 1.88, 95% CI 1.11–3.18, p = 0.03, model 3)
				All-cause mortality	↑ InCRP was associated with ↑ all-cause mortality (HR 1.19 1.05–1.34, p = 0.006, model 3). In cut-off: higher hsCRP levels (≥ 10 mg/L) vs. no inflammation levels (< 1 mg/L) were associated with ↑ all-cause mortality (HR 1.92, 95% CI 1.20–3.08, *p* = 0.006, model 3)

*Abbreviations*: *BMI* Body mass index, *CA 15–3* Cancer antigen 15–3, *CRP* C-reactive protein, *CV* Cardiovascular, *ER* Estrogen receptor, *FAM* Fully adjusted model, *HDL-c* High-density lipoprotein cholesterol, *HER2* Human epidermal growth factor receptor 2, *HOMA-IR* Homeostatic model assessment for insulin resistance, *HoR* Hormonal receptors, *HR* Hazard ratio, *hsCRP* high sensitivity C reactive protein, *HT* Hormonal therapy, *IGF-1* Insulin-like growth factor-1, *IGFBP-1* Insulin-like growth factor-binding protein 1, *IGFBP-3* Insulin-like growth factor-binding protein 3, *MetS* Metabolic syndrome, *PDGF* Platelet-derived growth factor, *PR* Progesterone receptor, *RCT* Randomized controlled trial, *RR* Risk of recurrence, *SHBG* Sex hormone-binding globulin, *T2D* Type 2 diabetes, *TG* Triglycerides, *WC* Waist circumference

^a^ All biomarkers were measured in fasting conditions

^b^ These variables were included in the models after assessing potential confounders. Berrino et al. [20] and Villaseñor et al. tested “intervention group” as a confounding variable; however it was not significant and therefore not included in the statistical models

^c^ 510 case-control pairs were analyzed for IGF-1, and a subgroup of 188 case-control pairs for IGFBP-1, IGFBP-3, leptin, adiponectin

No study was restricted to women in specific BMI categories or with certain metabolic diseases. However, DIANA-5 [23] reported that 419 women (20%) met the criteria for metabolic syndrome (MetS), and 897 women (42.9%) had a waist circumference (WC) ≥ 85 cm. Data from metabolomic signatures were not reported in any studies.

All studies included a range of estrogen receptor (ER) and progesterone receptor (PR) breast cancer subtypes [20–27]. DIANA-5 [23] and PACThe [24] recruited women with HER2+ breast cancer, while WHEL included women with HER2+ and HER2- subtypes [27].

DIANA-2 [20–22], DIANA-5 [23], and WHEL [26] analyzed blood biomarkers related to glucose, glucose metabolism and insulin resistance. Growth factors were measured in DIANA-2 [22] and WHEL [26]. Lipid profile biomarkers were reported in DIANA-2 [21], DIANA-5 [23], and PACThe [24], while sex hormones in DIANA-2 [20, 21], PACThe [24] and WHEL [25] were examined. Adipokines and inflammatory biomarkers were investigated in DIANA-2 [22] and WHEL [26, 27]. Table 4 presents the mean or median values of selected biomarkers, arranged by study and publication.

**Table 4:** Mean / median values of selected biomarkers, arranged by study

Authors (year) or study name, country	Patient population	Biomarker mean /median values
**DIANA-2**		
Berrino et al. (2005) [20]	Recurrent patients vs. non recurrent patients (all postmenopausal)	Mean values: Serum testosterone (0.52 vs. 0.38 ng/mL); Estradiol (8.06 vs. 5.52 pg/mL); Glucose (96 vs. 91 mg/dL). Data for insulin and SHBG were not reported
Pasanisi et al. (2006) [21]	Patients with MetS vs. patients without MetS (all postmenopausal)	Mean ± SD: Serum testosterone (0.49 ± 0.15 vs. 0.41 ± 0.15 ng/mL); Glucose (103 vs. 90.8 mg/dL); Insulin (12.4 ± 6.58 vs. 7.4 ± 3.0 μUI/mL); SHBG (46.3 ± 28.13 vs. 67.8 ± 29.75 nmol/L), Triglycerides (155.6 vs. 99.7 mg/dL); HDL-c (49.8 vs. 56.7 mg/dL)
Pasanisi et al. (2008) [22]	Recurrent patients vs. Non recurrent patients (all postmenopausal)	Mean ± SD: IGF-1 (188.2 ± 55.3 vs. 172.1 ± 60.3 ng/mL); PDGF (11.9 ± 5.1 vs. 9.4 ± 4.0 ng/mL); Fructosamine (329.1 ± 43.2 vs. 326.8 ± 48.7 μmol/L); CRP (1.97 ± 2.3 vs. 1.95 ± 2.9 mg/L)
**DIANA-5**		
Berrino et al. (2014) [23]	Patients with/without MetS (about 45% postmenopausal)	Means /ranges were not provided for any biomarker (glucose, HOMA-IR, triglycerides, HDL-c)
**PACThe study**		
Vasson et al. (2020) [24]	All patients (about 60% postmenopausal)	Mean ± SD: HDL-c (2.13 ± 1.28 mmol/L); Plasma testosterone (0.82 ± 0.36 nmol/L); CA 15–3 (18.1 ± 18.7 Ku/L)
**WHEL study**		
Emond et al. (2011) [25]	Comparison group, intervention group at baseline (all postmenopausal)	Median (IQR): Bioavailable estradiol (4.4 (2.8–7.1), 4.5 (2.8–7.1) pg/mL); Total estradiol (8.0 (5.0–12.0), 8.0 (5.0–12.0) pg/mL); Bioavailable testosterone (10.8 (7.4–16.2), 11.3 (7.8–15.2) ng/dL); Total testosterone (26.0 (19.3–37.8, 27.5 (20.8–37.3) ng/dL); SHBG (63.5 (44.0–91.5), 59.0 (43.3–90.0) nmol/L)
Al-Delaimy et al. (2011) [26]	Cases (recurred) vs. Controls (non-recurred) (about 75–80% postmenopausal)	Mean ± SEM: Insulin (320 [16] vs. 354 [23] pg/mL); Leptin (23,521 (1573) vs. 23,117 (1369) pg/mL); Adiponectin (9301 (334) vs. 9449 (365) ng/mL) IGF-1 (114.1 (2.3) vs. 108.3 (2.1) ng/mL); IGFBP-1 (32.0 (1.5) vs. 33.7 (1.8) ng/mL); IGFBP-3 (4.17 (0.07) vs. 4.21 (0.07) μg/mL)
Villaseñor et al. (2013) [27]	All patients (about 80% postmenopausal)	Median (IQR): Serum hsCRP (3.83 (0.67–4.24) mg/L)

*Abbreviations*: *CA 15–3* Cancer antigen 15–3, *HDL-c* High-density lipoprotein cholesterol, *HOMA-IR* Homeostatic model assessment for insulin resistance, *hsCRP* high sensitivity C-reactive protein, *IGF-1* Insulin-like growth factor-1, *IGFBP-3* Insulin-like growth factor-binding protein 3, *IQR* Interquartile range, *MetS* Metabolic syndrome, *SHBG* Sex hormone-binding globulin

Reference values for women: Serum testosterone: 8–60 ng/dL; serum estradiol: premenopausal: 15–350 pg/mL, postmenopausal: < 10 pg/mL; SHBG: aged 20 - 49y: 24.6–122 nmol/L, aged > 49 y: 19.3–76.4 nmol/L; fasting glucose: 70 - 140 mg/dL); fasting insulin: 2.6–24.9 μUl/mL; serum CA 15–3: < 30 U/mL;. HDL-c: ≥ 50 mg/dL; triglycerides: < 150 mg/dL; IGF-1: aged 31–35 years: 59–279 ng/mL, 36–40 years: 54–258 ng/mL, 41–45 years: 49–240 ng/mL, 46–50 years: 44–227 ng/mL, 51–55 years: 40–217 ng/mL, 56–60 years: 37–208 ng/mL; serum CRP: < 8 mg/L. (reference values retrieved on September 14, 2022 from https://www.mayocliniclabs.com/test-catalog/overview)

The reported prognosis outcomes were mainly the following: breast cancer recurrence in DIANA-2 [20–22], DIANA-5 [23], and WHEL [25–27], disease-free survival in PACThe [24], and breast cancer or all-cause mortality in WHEL [27]. Outcomes were described differently in each study. Table 5 provides a list of investigated outcomes per study. DIANA-2, WHEL, and PACThe carried out a mean/median follow-up period of ≥5 years [20–22, 24–27] while DIANA-5 had median follow-up period of 2.8 years [23].

**Table 5:** Definitions of outcomes for each study included in the systematic review

Authors (year) or study name, country	Outcomes	Outcomes definition
**DIANA-2**		
Berrino et al. (2005) [20]	Recurrence (local relapse, distant metastasis, contralateral breast cancer)	Local relapse, distant metastasis, contralateral breast cancer occurred over 5.5 years of follow-up
Pasanisi et al. (2006) [21]	Recurrence (local relapse, distant metastasis, contralateral breast cancer)	Local relapse, distant metastasis, contralateral breast cancer occurred over 5.5 years of follow-up
Pasanisi et al. (2008) [22]	Recurrence (local relapse, distant metastasis, contralateral breast cancer)	Local relapse, distant metastasis, contralateral breast cancer occurred over 5.5 years of follow-up
**DIANA-5**		
Berrino et al. (2014) [23]	Recurrence (loco-regional recurrences, distant metastasis and new primary breast cancer) and breast cancer mortality	Loco-regional recurrences, distant metastasis and new primary breast cancer, and deaths due to breast cancer occurred over a median of 2.8 years of follow-up
**PACThe study**		
Vasson et al. (2020) [24]	Disease-free survival (absence of local or distant (nodes, metastasis, and/or contralateral breast cancer))	Disease-free interval was computed as months elapsed from date of randomization to documented breast cancer recurrence during 7 years after breast cancer treatment completion. All recurrence types were considered, either local or distant (nodes, metastases, and/or contralateral breast cancer)
**WHEL study**		
Emond et al. (2011) [25]	Recurrence (local, regional, or distant, or new primary events)	Events were defined as local, regional, or distant recurrence, or new primary; the WHEL study did not include ductal carcinoma in situ or lobular carcinoma in situ as events. Time to outcome was date of randomization to date of additional event. Women who did not have an additional event were assessed for outcome at the date of last contact or study end (June 1, 2006). Women who died due to conditions unrelated to breast cancer were assessed for outcome at the date of death. WHEL further excluded women who had a breast cancer recurrence within the first year of study enrollment
Al-Delaimy et al. (2011) [26]	Breast cancer event (recurrent/new primary breast cancer event)	Reported breast cancer recurrence or new primary breast cancer diagnosed after study enrollment
Villaseñor et al. (2013) [27]	Additional breast cancer events (recurrence or new primary breast cancer) and breast cancer mortality	Women were followed during the follow-up for vital status, from study entry until end of study, June 1, 2006
	All-cause mortality	

### Associations between blood biomarkers and prognosis in breast cancer survivors

#### Testosterone

Associations between testosterone and breast cancer recurrence or disease-free survival were investigated in DIANA-2 [20, 21], PACThe [24] and WHEL [25]. Among DIANA-2 participants, women with breast cancer recurrence had higher serum testosterone (0.52 vs. 0.38 ng/ml, *p* < 0.001) [20]. When analyzed in tertiles, strong evidence was only found between the highest tertile of serum testosterone and an increased risk of recurrence (HR: 7.19, 95% CI 2.42–21.35, p not shown) [20] (Table 3). Moreover, women with MetS whose serum testosterone levels were > 0.40 ng/mL had a 6.7 times higher risk of recurrence compared with women without MetS whose serum testosterone levels were ≤ 0.40 ng/mL (HR: 6.7, 95% CI 2.3–19.8, p not shown) [21] (Table 3). Women without MetS whose serum testosterone levels were > 0.40 ng/mL showed a HR of 3.4 (95% CI 1.4–8.3), compared with the few women who had MetS and low serum testosterone levels [21].

Similar findings were reported in the WHEL study: Higher bioavailable testosterone levels were associated with a 69% increased risk of recurrence (HR for a one-unit increase in In-transformed values 1.69, 95% CI 1.00–2.84, *p* = 0.049) [25]. Sensitivity analyses showed that adjusting all models for baseline BMI did not impact the results, nor did limiting analyses to women without oophorectomies (*n* = 357) [25].

PACThe reported inverse associations between the lowest plasma testosterone levels and disease-free survival (*p* = 0.001) [24]. The Cox proportional hazards model suggested that testosterone was a prognostic marker, showing associations for the highest vs. lowest levels, with a ~ 5 fold increased risk of recurrence (HR 5.06, 95% CI 1.66–15.41, *p* = 0.004) (Table 3) [24]. Based on survival curves, testosterone was only relevant for disease-free survival in women treated with hormone therapy (*p* = 0.012 vs. *p* = 0.69, for women with and without hormone therapy, respectively) (Table 3) [24].

#### Estradiol and SHBG

Among DIANA-2 participants, although higher estradiol levels were observed in women with breast cancer recurrence compared to women without recurrence (8.06 vs. 5.52 pg/ml, *p* = 0.02), evidence was not found that elevated estradiol was associated with the risk of recurrence in hazards models adjusted for testosterone levels [20]. Likewise, no significant associations between estradiol levels and breast cancer recurrence were observed in the WHEL study [25]. Neither DIANA-2 [20] nor WHEL [25], the two studies which investigated SHBG, found evidence that this marker was associated with recurrence (Table 3).

#### Markers of glucose metabolism and insulin resistance

DIANA-2 reported that higher fasting plasma glucose levels were present in women with recurrence vs. women without recurrence (96 vs. 91 mg/dl, *p* = 0.02) [20] (Table 3). However, fasting glucose levels were not significantly associated with the risk of recurrence in this study [20, 21] nor in DIANA-5 [23]. Insulin levels were not associated with breast cancer events in the WHEL trial [26], nor with recurrence in DIANA-2 [20]. The DIANA-5 trial, which was the only study to assess the relationship between HOMA-IR, a marker of insulin resistance [28], and recurrence observed null associations [23].

#### Lipid profile

The DIANA-5 trial observed that lower HDL cholesterol (HDL-c) (OR 1.83, 95% CI 1.24–2.70) and higher triglyceride (TG) levels (OR 1.58, 95% CI 1.01–2.46, p not shown) were both associated with an increased risk for breast cancer recurrence [23]. After adjusting for other MetS characteristics, only lower HDL-c levels remained positively associated with risk for recurrence (OR 1.60, CI 95% 1.06–2.41) [23]. Similarly, the PACThe study observed that the highest HDL-c levels were associated with the longest survival without recurrence (*p* = 0.047) [24]. In contrast, DIANA-2 found no significant associations between either HDL-c or TG and breast cancer recurrence [21].

#### Growth factors

An observation made in the DIANA-2 cohort was that women with breast cancer recurrence presented with higher levels of platelet-derived growth factor (PDGF) vs. those without breast cancer recurrence (11.9 ng/mL vs. 9.4 ng/mL, respectively, *p* = 0.01) [22] (Table 3). Notably, neither IGF-1 nor PDGF alone was independently related to the risk for recurrence [22]. However, women who presented with higher levels of PDGF and IGF-1 combined (> 9.3 ng/mL and > 174.4 ng/mL, respectively) had a 6.4-fold increase in the risk for recurrence, in comparison to women with lower levels of PDGF and IGF-1 combined (highest vs. lowest quartile: HR 6.4, 95% CI, 1.5–26.7, p not shown) [22] (Table 3). The WHEL study did not observe any significant associations between IGF-1 and recurrence in the larger cohort (*n* = 510). Likewise, investigations with case-control pairs (*n* = 188) did not identify relationships between either insulin-like growth factor-binding protein (IGFBP)-1 or IGFBP-3 and recurrence [26].

#### Adipokines and inflammatory markers

WHEL was the only study to assess relationships between selected adipokines and breast cancer events, reporting null associations for both leptin and adiponectin (Table 3) [26]. Two studies that investigated associations between C-reactive protein (CRP) and breast cancer events reported inconsistent results [22, 27]. Higher InCRP levels in WHEL participants were significantly associated with an increased risk for additional breast cancer events (HR 1.13, 95% CI 1.03–1.24, *p* = 0.03, model 3) [27], while the DIANA-2 trial did not observe associations between CRP levels and recurrence [22]. Notably, in the WHEL study, the upper high-sensitivity CRP (hsCRP) levels (≥ 10 mg/L) were associated with a 65% increased risk of additional breast cancer events vs. no inflammation levels (< 1.0 mg/L) (HR 1.65, 95% CI 1.15–2.38, *p* = 0.03, full model adjusted for age, time since diagnosis, disease stage, disease grade, race–ethnicity, BMI, anti-estrogen use, and ER/PR status) [27]. The authors also observed that higher InCRP levels were associated with increased risk of both breast cancer- and all-cause mortality (HR 1.16 1.01–1.31, *p* = 0.03 and HR 1.19 1.05–1.34, *p* = 0.006, respectively, both from model 3) [27] (Table 3). Associations were even stronger among women with very high hsCRP levels (≥ 10 mg/L) vs. no inflammation levels (< 1.0 mg/L) (HR 1.88, 95% CI 1.11–3.18, *p* = 0.03, and HR 1.92, 95% CI 1.20–3.08, *p* = 0.006, respectively, both from model 3) [27] (Table 3).

Table 6 gives an overview of associations among obesity-related biomarker candidates and breast cancer prognosis outcomes reported in each study.

**Table 6:** Summary of associations between selected biomarkers and outcomes of breast cancer prognosis

	Testosterone	Estradiol/SHBG	Insulin/C-Peptide	HOMA-IR	HDL-c/TG	IGF-1/IGFBP-1, IGFBP-3/PDGF	Adipokines (Adiponectin/Leptin)	CRP
**Recurrence**	**↑** WHEL [25]	**—** Estradiol, SHBG DIANA 2 [20]; WHEL [25]	**—** Insulin DIANA 2 [20]	—DIANA 5 [23]	-- HDL-c, TG DIANA 2 [21] **↓** HDL-c DIANA 5 [23] **↑** TG DIANA 5 [23]	**—** IGF-1, PDGF DIANA 2 [22] **↑** IGF-1 + PDGF DIANA 2 [22]	N.A.	**—** DIANA 2 [22]
	**↑** DIANA 2 [20]							
	**↑** T + MetS^a^ DIANA 2 [21]							
	**↑** PACThe [24]							
**Disease-free survival**	**↓** PACThe [24]	N.A.	N.A.	N.A.	**↑** HDL-c PACThe [24]	N.A.	N.A.	N.A.
**Breast cancer event**	N.A.	N.A.	**—** Insulin WHEL [26]	N.A.	N.A.	**—** IGF-1, IGFBP-3 WHEL [26]	**—**Adiponectin, Leptin WHEL [26]	**↑** WHEL [27]
**Breast cancer mortality**	N.A.	N.A.	N.A.	N.A.	N.A.	N.A.	N.A.	**↑** WHEL [27]
**All-cause mortality**	N.A.	N.A.	N.A.	N.A.	N.A.	N.A.	N.A.	**↑** WHEL [27]

*Abbreviations*: *CRP* C-reactive protein, *HDL-c* High-density lipoprotein cholesterol, *HOMA-IR* Homeostatic model assessment for insulin resistance, *IGF-1* Insulin-like growth factor-1, *IGFBP-1* Insulin-like growth factor-binding protein 1, *IGFBP-3* Insulin-like growth factor-binding protein 3, *PDGF* Platelet-derived growth factor, *SHBG* Sex hormone-binding globulin, *TG* Triglycerides

**↑** significant positive association; **↓** significant negative association; —no significant association; N.A. not assessed

^a^ Women with metabolic syndrome and testosterone > 0.40 ng/mL

## Discussion

Circulating biomarkers could serve as prognostic indicators when assessing the efficacy of lifestyle intervention programs on survival. Hence, this systematic review was conducted to identify candidate obesity-related and other circulating biomarkers with prognostic value for female breast cancer survivors enrolled in lifestyle intervention trials.

In total, four studies that met the inclusion criteria were identified, highlighting the paucity of studies that investigate the predictive power of blood-borne biomarkers in this population. The range of biomarkers and heterogeneity of data precluded the ability to perform a meta-analysis.

Due to the epidemic of obesity in the adult population, a large proportion of women with breast cancer is overweight or obese. The risk of both breast cancer specific and total mortality are increased in women with overweight and obesity [3, 5, 29, 30]. This elevated risk is similarly observed in both pre- and postmenopausal women [5]. In addition, therapies in breast cancer survivors with obesity are less effective and associated with more complications compared with the non-obese population [4].

Obesity, per se, is characterized by a variety of metabolic and hormonal abnormalities, including subclinical inflammation, which may contribute to breast cancer progression [10, 31]. Excess adipose tissue not only increases estrogen production and hyperinsulinemia, but also produces a multitude of cytokines, growth, and angiogenic factors, all of which may foster tumor progression via various biological pathways. Findings from this review suggest that higher bioavailable and serum testosterone concentrations are linked to breast cancer recurrence, while lower plasma testosterone is associated with disease-free survival. These observations are in agreement with Roberts et al. [10], who reported a direct correlation between obesity and testosterone levels in women, and Coradini et al. [15] who observed that adipose tissue is involved in regulating the production and bioavailability of sex hormones, for instance in converting testosterone to estradiol via aromatase. In postmenopausal women with obesity, adipose tissue expression of aromatase may be increased and contribute to the enhanced production of testosterone and estradiol from precursors [32]. Moreover, the bioavailability of testosterone and estradiol is increased in obesity due to hyperinsulinemia and elevated IGF-1 activity, resulting in decreased hepatic production of SHBG [31]. However, this review did not uncover evidence that concentrations of either SHBG or estradiol were related to breast cancer recurrence [20, 25].

Although disturbances of glucose metabolism and insulin resistance seem to be connected to a greater risk of breast cancer as well as to a poorer prognosis [10, 33, 34], the studies included in this review did not show that these parameters could be relevant prognostic indicators in breast cancer patients. We found no evidence that glucose [20, 21, 23], insulin [20, 26] or HOMA-IR [23] were associated with recurrence.

Dysregulation of the insulin/IGF system, which is common in overweight and obesity, has been shown to influence breast cancer development via endocrine, paracrine, or autocrine signaling pathways [10, 35]. Circulating IGF-1 levels may be associated with a worse outcome, whereas elevated tissue IGF-1 levels seem to be protective [35]. Only about 1% of the circulating IGF-1 is free, with the overwhelming percentage bound to proteins (IGFBP), predominantly to IGFBP-3 [35]. Notably, the two studies in this review investigating associations between insulin-like growth factors and breast cancer prognosis reported inconsistent findings [22, 26]. Hence, data on these associations is limited and precludes the ability to draw conclusions on the role of growth factors in breast cancer progression.

Among the broad variety of factors released from adipose tissue, leptin and adiponectin are rather specific for adipose origin and interesting candidates for numerous reasons. Leptin is produced not only in adipose tissue, but also in cancer-associated adipocytes (CAAs), to an even greater extent than in mature adipocytes [36]. Moreover, leptin is secreted from cancer-associated fibroblasts (CAFs) which are located in the tumor microenvironment and continuously interact in crosstalk with breast cancer cells [36, 37]. Leptin has been described as fostering tumor development through the activation of various signaling pathways, by stimulating the proliferation and growth of breast cancer cells, and by promoting angiogenesis, invasion, and metastasis [36, 37]. Leptin also exerts proinflammatory effects [31] and can render breast cancer cells less susceptible to treatment with tamoxifen [38]. Elevated levels of leptin were observed in higher grade and advanced tumor stages of breast cancer [36, 37] and were associated with a poorer outcome in triple-negative breast cancer [39]. Furthermore, a meta-analysis demonstrated a positive association between leptin levels and the incidence of breast cancer, with the strongest evidence seen in postmenopausal women with overweight/obesity [40].

Evidence suggests that adiponectin confers protective effects against breast cancer progression [37] by activating AMP-Kinase and suppressing PI3K/AKT signaling, thereby inhibiting tumor growth, angiogenesis, and invasion and by inducing cell apoptosis [36, 37]. Adiponectin also exhibits anti-inflammatory properties [31]. A meta-analysis investigating the association between adiponectin levels and breast cancer risk found that serum adiponectin was lower in women developing breast cancer versus controls, irrespective of menopausal status [41]. While we did not find evidence that leptin or adiponectin concentrations measured after primary breast cancer treatment can predict tumor progression, the data are scarce, as adipokines were only examined in one study included in this review [26].

The low-grade inflammation of adipose tissue found in obesity is characterized by the abnormal production of cytokines, an elevated synthesis of acute-phase proteins such as CRP, and the activation of pro-inflammatory signaling pathways [10]. Elevated CRP, used as a surrogate marker for inflammation, was reported to be associated with many types of primary operable cancers [42, 43]. In our review, the evidence for prognostic relevance of circulating levels of CRP was too weak to draw conclusions [27]. Although significant associations have been reported by others [43], it is important to note that the clinical relevance of CRP as a predictive biomarker is questionable since elevated levels are observed in many diseases.

It is biologically plausible that intentional weight loss can lead to improvements in several obesity-related biomarkers. Ongoing studies are evaluating the beneficial effects of lifestyle interventions on recurrence and survival, including changes in associated circulating markers [6, 44, 45]. Hence, integrating lifestyle intervention programs aimed at intentional weight loss or weight stabilization, as appropriate, into the standard clinical treatment plan for breast cancer survivors cannot be overemphasized. Although our review uncovered limited evidence, these findings provide a starting point from which researchers designing lifestyle intervention programs can further explore the clinical utility of selected prognostic blood biomarkers.

### Strengths and limitations

This study represents a comprehensive and systematic review of randomized controlled lifestyle intervention studies investigating associations between baseline circulating biomarkers and prognosis in breast cancer survivors. These findings are only applicable to breast cancer survivors with no evidence of disease, thus generalizability is limited. Moreover, biomarkers were only assessed at one time point. Hence, we were unable to explore relationships between changes in biomarker levels and prognostic outcomes. A severe limitation is the heterogeneity of studies included in this review, whose populations differed in size, ethnic background, and other characteristics, including menopausal status and treatment with adjuvant hormonal therapy. A future direction of research that could be applied to overcome some limitations and provide greater insights into these relationships is a meta-analysis of individual participant data. However, this approach would necessitate the standardization of some study characteristics, such as study population, study design, follow-up length and timing of biomarker measurement to allow for a meta-analysis of aggregate data.

Chemotherapy is shown to induce metabolic alterations, including changes in lipid- [46] and glucose metabolism [47], which may lead to insulin resistance [46]. That could result in confounding of the relationships for associated biomarkers. Therefore, to minimize bias resulting from chemotherapy, studies that included participants undergoing chemotherapy during blood collection were not considered. All studies reported that women were disease-free when recruited into the lifestyle intervention trials. However, we included studies with women who were undergoing hormonal therapy at the time that baseline biomarkers were measured. We are aware that hormonal treatment, e.g. tamoxifen, has been shown to influence concentrations of sex hormones and therefore our findings should be interpreted with caution [48]. However, this population was included because adjuvant hormonal therapy continues on average for 5 years, meaning that most of the published studies drew blood samples when women were undergoing treatment. Furthermore, although women from all BMI categories were included in most studies, DIANA-2, DIANA-5 and PACThe did not adjust for BMI in statistical models, while WHEL only adjusted for BMI in some analyses. Hence, the studies included in this review did not consistently consider the modifying role of BMI when assessing relationships between biomarkers and breast cancer prognosis.

The included RCTs pooled intervention and control groups to investigate outcomes of interest. We cannot rule out that biomarkers measured in the intervention and control groups at baseline would have had differential relationships with prognosis. Studies considered this bias in different ways. For instance, Berrino et al. from the DIANA-2 trial assessed intervention group as a potential confounder. However, they did not include group allocation in their final statistical models after observing that it was not significant [20]. They also note that hormone levels between the two groups at baseline and 1-year post-intervention were largely similar, supporting their decision to pool groups in subsequent analyses. WHEL adjusted for intervention group in one study [25], and determined that adjustment for this confounder did not change the risk estimates for CRP in another analysis [27]. It is doubtful that the PACThe intervention had lasting effects on biological markers, as the intervention consisted of a 2-week diet and exercise program. Moreover, significant group differences in biomarkers and body weight were not observed 1 year post-intervention in the PACThe cohort [24]. Notably, most studies investigated obesity-related biomarkers as secondary analyses. Hence, the studies may have been statistically underpowered to detect associations between biomarkers and survival outcomes.

Despite efforts to identify novel biomarkers that may be clinically useful, there are currently no validated obesity-related circulating markers that predict recurrence or mortality for breast cancer survivors. Importantly, there is generally no consensus on the standard ranges for most of these biomarkers in breast cancer research, hence the definitions of “increased” or “decreased” concentrations are arbitrary. As described in this review, some studies defined a concentration above or below a certain threshold, such as the highest or lowest tertiles, whereas others investigated linear associations between biomarkers and disease progression.

## Conclusion

Evidence from studies included in this review suggests that bioavailable or serum testosterone may be positively linked to breast cancer recurrence, and that plasma testosterone may be inversely linked to disease-free survival. Overall, despite potential candidates, there is currently insufficient evidence to deduce that obesity-related and other circulating biomarkers have a predictive value for breast cancer prognosis. Several inconsistencies can be explained by the heterogeneity across studies and highlight the need for more large-scale, longitudinal studies that evaluate the clinical value of circulating biomarkers for predicting breast cancer progression and survival.

## Supplementary Information

**Additional file 1.** PRISMA 2020 Checklist.

**Additional file 2.** Search Strategy.

**Additional file 3.** Full-text articles excluded, with reasons (*n* = 84).

## Data Availability

All data generated or analyzed during this study are included in this published article (and its additional files).

## Abbreviations

BMI: Body mass index
CRP: C-reactive protein
CV: Cardiovascular
ER: Estrogen receptor
HDL: High-density lipoprotein
HER2: Human epidermal growth factor receptor 2
HOMA-IR: Homeostatic model assessment for insulin resistance
HoR: Hormonal receptors
HR: Hazard ratio
hsCRP: High sensitivity reactive protein
HT: Hormonal therapy
IGF-1: Insulin-like growth factor-1
IGFBP-3: Insulin-like growth factor-binding protein 3
MetS: Metabolic syndrome
NOS: Newcastle-Ottawa scale
OR: Odds ratio
PDGF: Platelet derived growth factor
PICOS/PECOS framework: Participants, intervention/exposure, control, outcome, study design
PR: Progesterone receptor
PRISMA: Preferred Reporting Items for Systematic Reviews and Meta-Analyses guidelines
PROSPERO: Prospective Register of Systematic Reviews
RCT: Randomized controlled trial
SHBG: Sex hormone binding globulin
T2D: Type 2 diabetes
