# Supplementary material for: Associations between circulating obesity-related biomarkers and prognosis in female breast cancer survivors: a systematic review of observational data in women enrolled in lifestyle intervention trials
Source: BMC Cancer. 2022 Nov 18;22:1187. doi: 10.1186/s12885-022-10274-3 (PMC9673384; doi:10.1186/s12885-022-10274-3)
Supplement: Supplementary file 2 — Additional file 2. Search Strategy. [file 12885_2022_10274_MOESM2_ESM.pdf]

Search Strategy for Meyer, et al.

OBSERVATIONAL STUDIES

EMBASE

URL: https://www.embase.com/login#advancedSearch/default

Date: 26.08.20

Keywords:

('Breast tumor '/exp OR ‘breast neoplas*’:ti,ab,kw OR ‘breast cancer*’:ti,ab,kw OR ‘breast tumor*’:ti,ab,kw OR ‘breast carcinoma*’:ti,ab,kw OR ‘mammary cancer*’:ti,ab,kw OR ‘mammary tumor*’:ti,ab,kw OR ‘mammary neoplas*’:ti,ab,kw OR ‘mammary carcinoma*’:ti,ab,kw) AND ('Cancer survivor '/exp OR survivor*:ti,ab,kw OR 'Aftercare '/exp OR aftercare:ti,ab,kw OR 'Recurrent disease '/exp OR recurrence*:ti,ab,kw) AND ('Biological marker'/exp OR biomarker*:ti,ab,kw OR ‘biological marker*’:ti,ab,kw OR ‘serum marker*’:ti,ab,kw OR ‘clinical marker*’:ti,ab,kw OR ‘plasma marker*’:ti,ab,kw OR ‘blood marker*’:ti,ab,kw OR 'Insulin '/exp OR insulin:ti,ab,kw OR glucose:ti,ab,kw OR 'Cytokine '/exp OR cytokine*:ti,ab,kw OR adipokine*:ti,ab,kw OR adipocytokine*:ti,ab,kw OR 'leptin '/exp OR leptin:ti,ab,kw OR adiponectin:ti,ab,kw OR resistin:ti,ab,kw OR cardiovascular:ti,ab,kw OR ‘metabolic profile*’:ti,ab,kw OR 'Metabolomics '/exp OR metabolomic*:ti,ab,kw) AND ('Recurrent disease '/exp OR recurrence*:ti,ab,kw OR 'Prognosis '/exp OR prognos*:ti,ab,kw OR 'Mortality '/exp OR mortality:ti,ab,kw OR 'survival '/exp OR survival:ti,ab,kw) AND ('Body Weight '/exp OR ‘body weight’ OR overweight OR 'obesity '/exp OR obes* OR adipo* OR 'Body Mass '/exp OR ‘body mass index’ OR 'Metabolic Syndrome X '/exp OR ‘metabolic syndrome’ OR 'Inflammation '/exp OR ‘inflammat*’)

Filters used: none

Reference count: 2012

PubMed

URL: https://www.ncbi.nlm.nih.gov/pubmed/advanced

Date: 26.08.20

Keywords:

(Breast Neoplasms [Mesh] OR breast neoplas* [Title/Abstract] OR breast cancer* [Title/Abstract] OR breast tumor* [Title/Abstract] OR breast carcinoma* [Title/Abstract] OR mammary cancer* [Title/Abstract] OR mammary tumor* [Title/Abstract] OR mammary neoplas* [Title/Abstract] OR mammary carcinoma* [Title/Abstract] OR breast neoplas* [Text Word] OR breast cancer* [Text Word] OR breast tumor* [Text Word] OR breast carcinoma* [Text Word] OR mammary cancer* [Text Word] OR mammary tumor* [Text Word] OR mammary neoplas* [Text Word] OR mammary carcinoma* [Text Word]) AND (Cancer survivors [Mesh] OR survivor* [Title/Abstract] OR survivor* [Text Word] OR Aftercare [Mesh] OR aftercare [Title/Abstract] OR aftercare [Text Word] OR Recurrence [Mesh] OR recurrence* [Title/Abstract] OR recurrence* [Text Word]) AND (Biomarkers [Mesh] OR biomarker* [Title/Abstract] OR biological marker* [Title/Abstract] OR serum marker* [Title/Abstract] OR clinical marker* [Title/Abstract] OR plasma marker* [Title/Abstract] OR blood marker* [Title/Abstract] OR biomarker* [Text Word] OR biological marker* [Text Word] OR serum marker* [Text Word] OR clinical marker* [Text Word] OR plasma marker* [Text Word] OR blood marker* [Text Word] OR Insulin [Mesh]

OR insulin [Title/Abstract] OR insulin [Text Word] OR glucose [Title/Abstract] OR glucose [Text Word] OR Adipokines [Mesh] OR adipokine* [Title/Abstract] OR adipocytokine* [Title/Abstract] OR leptin [Title/Abstract] OR adiponectin [Title/Abstract] OR resistin [Title/Abstract] OR adipokine* [Text Word] OR adipocytokine* [Text Word] OR leptin [Text Word] OR adiponectin [Text Word] OR resistin [Text Word] OR Cytokines [Mesh] or cytokine* [Title/Abstract] OR cardiovascular [Title/Abstract] OR metabolic profile* [Title/Abstract] OR Metabolomics [Mesh] OR metabolomic* [Title/Abstract] OR cytokine* [Text Word] OR cardiovascular [Text Word] OR metabolic profile* [Text Word] OR metabolomic* [Text Word]) AND (Recurrence [Mesh] OR recurrence* [Title/Abstract] OR Prognosis [Mesh] OR prognos* [Title/Abstract] OR Mortality [Mesh] OR mortality [Title/Abstract] OR survival [Mesh] OR survival [Title/Abstract] OR recurrence* [Text Word] OR prognos* [Text Word] OR mortality [Text Word] OR survival [Text Word]) AND (Body Weight [Mesh] OR body weight [all] OR overweight [all] OR obes* [all] OR adipo* [all] OR Body Mass Index [Mesh] OR body mass index [all] OR Metabolic Syndrome [Mesh] OR metabolic syndrome [all] OR Inflammation [Mesh] OR inflammat* [all])

Filters used: None

Reference count: 654

COCHRANE LIBRARY

URL: https://www.cochranelibrary.com/advanced-search/search-manager

Date: 26.08.20

keywords:

(MeSH descriptor: [Breast Neoplasms] explode all trees OR (breast neoplas*):ti,ab,kw OR (breast cancer*):ti,ab,kw OR (breast tumor*):ti,ab,kw OR (breast carcinoma*):ti,ab,kw OR (mammary cancer*):ti,ab,kw OR (mammary tumor*):ti,ab,kw OR (mammary neoplas*):ti,ab,kw OR (mammary carcinoma*):ti,ab,kw) AND (MeSH descriptor: [Cancer Survivors] explode all trees OR (survivor*):ti,ab,kw OR MeSH descriptor: [Recurrence] explode all trees OR (recurrence*):ti,ab,kw OR MeSH descriptor: [Aftercare] explode all trees OR (aftercare):ti,ab,kw) AND (MeSH descriptor: [Biomarkers] explode all trees OR (biomarker*):ti,ab,kw OR (biological marker*):ti,ab,kw OR (serum marker*):ti,ab,kw OR (clinical marker*):ti,ab,kw OR (plasma marker*):ti,ab,kw OR (blood marker*):ti,ab,kw OR MeSH descriptor: [Insulins] explode all trees OR (insulin):ti,ab,kw OR (glucose):ti,ab,kw OR MeSH descriptor: [Adipokines] explode all trees OR (adipokine*):ti,ab,kw OR (adipocytokine*):ti,ab,kw OR (leptin):ti,ab,kw OR (adiponectin):ti,ab,kw OR (resistin):ti,ab,kw OR MeSH descriptor: [Cytokines] explode all trees OR (cytokine):ti,ab,kw OR (cardiovascular):ti,ab,kw OR (metabolic profile*):ti,ab,kw OR MeSH descriptor: [Metabolomics] explode all trees OR (metabolomic*):ti,ab,kw) AND (MeSH descriptor: [Recurrence] explode all trees OR (recurrence):ti,ab,kw OR MeSH descriptor: [Prognosis] explode all trees OR (Prognos*):ti,ab,kw OR MeSH descriptor: [Mortality] explode all trees OR (mortality):ti,ab,kw OR MeSH descriptor: [Survival] explode all trees OR (survival):ti,ab,kw) AND (MeSH descriptor: [Body Weight] explode all trees OR (body weight):ti,ab,kw OR (overweight):ti,ab,kw OR (obes*):ti,ab,kw OR (adipo*):ti,ab,kw OR MeSH descriptor: [Body Mass Index] explode all trees OR (body mass index):ti,ab,kw OR MeSH descriptor: [Metabolic Syndrome] explode all trees OR (metabolic syndrome):ti,ab,kw OR MeSH descriptor: [Inflammation] explode all trees OR (inflammat*):ti,ab,kw)

Filters used: none

Reference count: 260

INTERVENTION STUDIES

EMBASE

URL: https://www.embase.com/login#advancedSearch/default

Date: 26.08.20

Keywords:

('Breast tumor '/exp OR "breast neoplas*":ti,ab,kw OR "breast cancer*":ti,ab,kw OR "breast tumor*":ti,ab,kw OR "breast carcinoma*":ti,ab,kw OR "mammary cancer*":ti,ab,kw OR "mammary tumor*":ti,ab,kw OR "mammary neoplas*":ti,ab,kw OR "mammary carcinoma*":ti,ab,kw) AND ('Cancer survivor '/exp OR survivor*:ti,ab,kw OR 'Aftercare '/exp OR aftercare:ti,ab,kw OR 'Recurrent disease '/exp OR recurrence*:ti,ab,kw) AND ('Biological marker'/exp OR biomarker*:ti,ab,kw OR "biological marker*":ti,ab,kw OR "serum marker*":ti,ab,kw OR "clinical marker*":ti,ab,kw OR "plasma marker*":ti,ab,kw OR "blood marker*":ti,ab,kw OR 'Insulin '/exp OR insulin:ti,ab,kw OR glucose:ti,ab,kw OR 'Cytokine '/exp OR cytokine*:ti,ab,kw OR adipokine*:ti,ab,kw OR adipocytokine*:ti,ab,kw OR 'leptin '/exp OR leptin:ti,ab,kw OR adiponectin:ti,ab,kw OR resistin:ti,ab,kw OR cardiovascular:ti,ab,kw OR "metabolic profile*":ti,ab,kw OR 'Metabolomics '/exp OR metabolomic*:ti,ab,kw) AND ('Caloric Restriction '/exp OR "caloric restriction*":ti,ab,kw OR 'Exercise '/exp OR exercis*:ti,ab,kw OR 'physical activity '/exp OR "physical activity":ti,ab,kw OR 'Diet '/exp OR diet*:ti,ab,kw OR 'diet therapy '/exp OR nutrition*:ti,ab,kw OR 'body weight loss '/exp OR "weight loss":ti,ab,kw OR "weight change*":ti,ab,kw OR "weight reduction*":ti,ab,kw OR 'Lifestyle '/exp OR lifestyle:ti,ab,kw OR 'health behavior '/exp OR behavior*:ti,ab,kw)

Filters used: none

Reference #: 1732

PubMed

URL: https://www.ncbi.nlm.nih.gov/pubmed/advanced

Date: 26.08.20

Keywords:

(Breast Neoplasms [Mesh] OR breast neoplas* [Title/Abstract] OR breast cancer* [Title/Abstract] OR breast tumor* [Title/Abstract] OR breast carcinoma* [Title/Abstract] OR mammary cancer* [Title/Abstract] OR mammary tumor* [Title/Abstract] OR mammary neoplas* [Title/Abstract] OR mammary carcinoma* [Title/Abstract] OR breast neoplas* [Text Word] OR breast cancer* [Text Word] OR breast tumor* [Text Word] OR breast carcinoma* [Text Word] OR mammary cancer* [Text Word] OR mammary tumor* [Text Word] OR mammary neoplas* [Text Word] OR mammary carcinoma* [Text Word]) AND (Cancer survivors [Mesh] OR survivor* [Title/Abstract] OR survivor* [Text Word] OR Aftercare [Mesh] OR aftercare [Title/Abstract] OR aftercare [Text Word] OR Recurrence [Mesh] OR recurrence* [Title/Abstract] OR recurrence* [Text Word]) AND (Biomarkers [Mesh] OR biomarker* [Title/Abstract] OR biological marker* [Title/Abstract] OR serum marker* [Title/Abstract] OR clinical

marker* [Title/Abstract] OR plasma marker* [Title/Abstract] OR blood marker* [Title/Abstract] OR biomarker* [Text Word] OR biological marker* [Text Word] OR serum marker* [Text Word] OR clinical marker* [Text Word] OR plasma marker* [Text Word] OR blood marker* [Text Word] OR Insulin [Mesh] OR insulin [Title/Abstract] OR insulin [Text Word] OR glucose [Title/Abstract] OR glucose [Text Word] OR Adipokines [Mesh] OR adipokine* [Title/Abstract] OR adipocytokine* [Title/Abstract] OR leptin [Title/Abstract] OR adiponectin [Title/Abstract] OR resistin [Title/Abstract] OR adipokine* [Text Word] OR adipocytokine* [Text Word] OR leptin [Text Word] OR adiponectin [Text Word] OR resistin [Text Word] OR Cytokines [Mesh] or cytokine* [Title/Abstract] OR cardiovascular [Title/Abstract] OR metabolic profile* [Title/Abstract] OR Metabolomics [Mesh] OR metabolomic* [Title/Abstract] OR cytokine* [Text Word] OR cardiovascular [Text Word] OR metabolic profile* [Text Word] OR metabolomic* [Text Word]) AND (Caloric Restriction [Mesh] OR caloric restriction* [Title/Abstract] OR Exercise [Mesh] OR exercis* [Title/Abstract] OR physical activity [Title/Abstract] OR Diet [Mesh] OR diet* [Title/Abstract] OR nutrition therapy [Mesh] OR nutrition* [Title/Abstract] OR Weight Loss [Mesh] OR weight loss [Title/Abstract] OR weight change* [Title/Abstract] OR weight reduction* [Title/Abstract] OR Life style [Mesh] OR lifestyle [Title/Abstract] OR health behavior [Mesh] OR behavior* [Title/Abstract] OR caloric restriction* [Text Word] OR exercis* [Text Word] OR physical activity [Text Word] OR diet* [Text Word] OR nutrition* [Text Word] OR weight loss [Text Word] OR weight change* [Text Word] OR weight reduction* [Text Word] OR lifestyle [Text Word] OR behavior* [Text Word])

Filters used: None

Reference #: 793

COCHRANE LIBRARY

URL: https://www.cochranelibrary.com/advanced-search/search-manager

Date: 26.08.20

Keywords:

(MeSH descriptor: [Breast Neoplasms] explode all trees OR (breast neoplas*):ti,ab,kw OR (breast cancer*):ti,ab,kw OR (breast tumor*):ti,ab,kw OR (breast carcinoma*):ti,ab,kw OR (mammary cancer*):ti,ab,kw OR (mammary tumor*):ti,ab,kw OR (mammary neoplas*):ti,ab,kw OR (mammary carcinoma*):ti,ab,kw) AND (MeSH descriptor: [Cancer Survivors] explode all trees OR (survivor*):ti,ab,kw OR MeSH descriptor: [Aftercare] explode all trees OR (aftercare):ti,ab,kw OR MeSH descriptor: [Recurrence] explode all trees OR (recurrence*):ti,ab,kw) AND (MeSH descriptor: [Biomarkers] explode all trees OR (biomarker*):ti,ab,kw OR (biological marker*):ti,ab,kw OR (serum marker*):ti,ab,kw OR (clinical marker*):ti,ab,kw OR (plasma marker*):ti,ab,kw OR (blood marker*):ti,ab,kw OR MeSH descriptor: [Insulins] explode all trees OR (insulin):ti,ab,kw OR (glucose):ti,ab,kw OR MeSH descriptor: [Adipokines] explode all trees OR (adipokine*):ti,ab,kw OR (adipocytokine*):ti,ab,kw OR (leptin):ti,ab,kw OR (adiponectin):ti,ab,kw OR (resistin):ti,ab,kw OR MeSH descriptor: [Cytokines] explode all trees OR (cytokine):ti,ab,kw OR (cardiovascular):ti,ab,kw OR (metabolic profile*):ti,ab,kw OR MeSH descriptor: [Metabolomics] explode all trees OR (metabolomic*):ti,ab,kw) AND (MeSH descriptor: [Caloric Restriction] explode all trees OR (caloric restriction*):ti,ab,kw OR MeSH descriptor: [Exercise] explode all trees OR (exercis*):ti,ab,kw OR (physical activity):ti,ab,kw OR MeSH descriptor: [Diet] explode all trees OR (diet*):ti,ab,kw MeSH descriptor: [Nutrition Therapy] explode all trees OR (nutrition*):ti,ab,kw OR

MeSH descriptor: [Weight Loss] explode all trees OR (weight loss):ti,ab,kw OR (weight change*):ti,ab,kw OR (weight reduction*):ti,ab,kw OR MeSH descriptor: [Life Style] explode all trees OR (lifestyle):ti,ab,kw OR MeSH descriptor: [Health Behavior] explode all trees OR (behavior*):ti,ab,kw)

Filters used: none

Reference #: 316
