# Supplementary material for: Associations between circulating obesity-related biomarkers and prognosis in female breast cancer survivors: a systematic review of observational data in women enrolled in lifestyle intervention trials
Source: BMC Cancer. 2022 Nov 18;22:1187. doi: 10.1186/s12885-022-10274-3 (PMC9673384; doi:10.1186/s12885-022-10274-3)
Supplement: Supplementary file 3 — Additional file 3. Full-text articles excluded, with reasons (n = 84). [file 12885_2022_10274_MOESM3_ESM.pdf]

Meyer, et al. Additional File 3: Full-text articles excluded, with reasons (n=84)

- No control group of interest or no true control arm (n= 7)
- No exposure of interest (n= 4)
- No intervention of interest (n=4)
- No outcome of interest (n= 8)
- Non-randomized study (n=5)
- Other (n=2)
- Narrative reviews (n=2)
- Wrong population (n=18)
- Study protocol (n=27)
- Foreign language (n=2)
- Data not available (n=1)
- Wrong study design (n=4)

(Adlard et al., 2019)	Study protocol
(Alfano et al., 2017)	No outcome of interest
(Arikawa, Kaufman, Raatz, & Kurzer, 2018)	No true control arm
(Augustin et al., 2017)	Wrong population – non breast cancer survivors
(Azamian, Mobarekeh, Vismeh, & Gohar, 2015)	Paper in Arabic
(Bahl et al., 2005)	Wrong population – did not fulfill inclusion criteria
(Befort et al., 2014)	Study protocol
(Bera et al., 2020a)	Other – Corrigendum to: Proteomic Analysis of Inflammatory Biomarkers Associated With Breast Cancer Recurrence
(Bera et al., 2020b)	Data not available – no information on breast cancer stage or timing of blood draw
(Blackburn & Wang, 2007)	Wrong population - did not fulfill inclusion criteria
(Bobin-Dubigeon, Lefrançois, Classe, Joalland, & Bard, 2015)	Wrong population - did not fulfill inclusion criteria
(Bower et al., 2014)	No true control arm
(Bramwell et al., 2014)	Wrong population - did not fulfill inclusion criteria
(Calip et al., 2014)	Wrong population - did not fulfill inclusion criteria
(Cormie et al., 2016)	No intervention of interest
(Dittus et al., 2018)	No outcome of interest
(Duggan et al., 2011)	Wrong study design
(Duggan et al., 2013)	Wrong study design
(Fujii et al., 2014)	Wrong population - did not fulfill inclusion criteria

(Furberg, Veierød, Wilsgaard, Bernstein, & Thune, 2004)	Wrong population - did not fulfill inclusion criteria
(Galanti, Stefani, & Gensini, 2013)	Other- description of exercise program for Breast cancer survivors
(Gentry et al., 2018)	Study protocol
(Ghosh, Hughes, Parma, Ramirez, & Li, 2014)	No outcome of interest
(Giganti et al., 2016)	No outcome of interest
(Gnagnarella et al., 2016)	Study protocol
(Goodwin et al., 2002)	Wrong population – did not fulfill inclusion criteria
(Hutnick et al., 2005)	Non-randomized study
(M. R. Irwin et al., 2014)	No intervention of interest –Tai chi study
(M. L. Irwin et al., 2011)	Wrong study design
(Janelsins et al., 2011)	No true control arm
(Johansen, Cintin, Jørgensen, Kamby, & Price, 1995)	Wrong population - did not fulfill inclusion criteria
(Jones et al., 2010)	No outcome of interest
(Knobf & Coviello, 2011)	Narrative review
(Kus, Cinkir, Aktas, & Abali, 2019)	No exposure of interest
(Larkey, Huberty, Pedersen, & Weihs, 2016)	No intervention of interest – Qigong, Tai chi study
(Long Parma et al., 2015)	No control arm
(McEligot et al., 1999)	No outcome of interest
(Meyerhardt et al., 2020)	Wrong population – cohort were breast cancer and colon cancer survivors
(Nelson et al., 2017)	Wrong population - did not fulfill inclusion criteria
(Nock et al., 2013)	Non-randomized study
(Nuri et al., 2014)	Paper in Arabic
(Oh et al., 2011)	Wrong population - did not fulfill inclusion criteria
(Owusu et al., 2020)	Study protocol
(Pierce et al., 2009)	Wrong study design
(Pudkasam et al., 2020)	Study protocol
(Ramirez et al., 2017)	Study protocol
(Reeves et al., 2016)	Study protocol
(Repka & Hayward, 2018)	Wrong population – did not fulfill inclusion criteria
(Rhone, Bielawski, Ziołkowska, Rość, & Ruszkowska-Ciastek, 2019)	No exposure of interest
(Rock et al., 2013)	No control group of interest
(Rock et al., 2008)	Study protocol
(Rothammer, Sage, Werner, Combs, & Multhoff, 2019)	Wrong population - did not fulfill inclusion criteria
(Sestak, Dowsett, Ferree, Baehner, & Cuzick, 2016)	No exposure of interest

(Skouroliakou et al., 2018)	Wrong population - did not fulfill inclusion criteria
(Sprod et al., 2012)	No intervention of interest – Qigong study
(Stoll, 1996)	Narrative review
(Stolley et al., 2017)	No outcome of interest
(Študentová et al., 2015)	No exposure of interest
(Thomson et al., 2010)	No true control arm – parallel arm study
(Walter et al., 2019)	Non-randomized study
(Zhang et al., 2019)	Wrong population – patients with Stage IV breast cancer were included
12-week Exercise Intervention Program Versus Observation in Early Stage Breast Cancer Patients on the Impact on Mental Health, Quality of Life and Immune Markers https://clinicaltrials.gov/show/NCT03518957	Study protocol
A peer support program for the long-term maintenance of physical activity and health outcomes in breast, prostate and colorectal cancer survivors http://www.who.int/trialsearch/Trial2.aspx?TrialID=ACTRN12618001855213	Study protocol
Avanzando Juntas: adapting an Evidence Based Weight Loss Program for Hispanic Breast Cancer Survivors https://clinicaltrials.gov/show/NCT04321135	Study protocol
Combined diet and exercise intervention among breast cancer survivors https://www.cochranelibrary.com/central/doi/10.1002/central/CN-01824672/full	Non-randomized study, Clinical trial register
Developing a Healthy Lifestyle in Breast Cancer Survivors https://clinicaltrials.gov/show/NCT02677857	Study protocol
Development of exercise training regimen as second prevention for breast cancer survivors http://www.who.int/trialsearch/Trial2.aspx?TrialID=JPRN-UMIN000013475	Study protocol
Diet Composition, Weight Control, and Breast Carcinogenesis https://clinicaltrials.gov/show/NCT01315483	Non-randomized study, Clinical trial register
Exercise in Improving Health and Quality of Life in Breast Cancer Survivors https://clinicaltrials.gov/show/NCT03679559	Study protocol
EXERCISING TOGETHER© for Couples Coping With Cancer https://clinicaltrials.gov/show/NCT03630354	Study protocol
Impact of Diet and Physical Activity Changes on Body Weight, Biomarkers and Quality of Life in Breast Cancer Survivors https://clinicaltrials.gov/show/NCT04096469	Study protocol
Impact of Weight Loss Interventions for Overweight Breast Cancer Survivors https://www.cochranelibrary.com/central/doi/10.1002/central/CN-01534191/full	No outcome of interest, Clinical trial register
Part II: exercise in Hispanic Breast Cancer Survivors https://clinicaltrials.gov/show/NCT01504789	Study protocol
Peer Counseling for Weight Loss https://clinicaltrials.gov/show/NCT00120029	Study protocol
Physical activity adherence, psychological health and immunological outcomes (PAPHIO study) in breast cancer survivors http://www.who.int/trialsearch/Trial2.aspx?TrialID=ACTRN12619001271190	Study protocol

Physical activity in Breast Cancer survivors: effects on physical capacity and quality of life http://www.who.int/trialsearch/Trial2.aspx?TrialID=RBR-3fw9xf	Study protocol
Promoting weight loss through diet and exercise in overweight women with breast cancer http://www.who.int/trialsearch/Trial2.aspx?TrialID=ISRCTN53325751	Study protocol
Rx for Better Breast Health https://clinicaltrials.gov/show/NCT02279303	Study protocol
The Breast Cancer Personalized Nutrition Study https://clinicaltrials.gov/show/NCT04079270	Study protocol
The effect of a long-term period interval and continuous exercise on some markers and inflammatory cytokines in breast cancer survivors http://www.who.int/trialsearch/Trial2.aspx?TrialID=IRCT20190731044398N2	Study protocol
The MATCH Study: mindfulness And Tai Chi for Cancer Health https://clinicaltrials.gov/show/NCT02801123	Study protocol
Using Behavioral Economics to Achieve Improved Healthy Behavior Outcomes in Breast Cancer Survivors https://clinicaltrials.gov/show/NCT02938780	Study protocol
Weight Gain Prevention for Breast Cancer Survivors https://www.cochranelibrary.com/central/doi/10.1002/central/CN-01577018/full	Wrong population - did not fulfill inclusion criteria – Clinical trial register
Weight Loss Pilot Study in Postmenopausal Breast Cancer Survivors https://clinicaltrials.gov/show/NCT02940470	No true control arm – parallel arm study, clinical trial register

References

Adlard, K. N., Jenkins, D. G., Salisbury, C. E., Bolam, K. A., Gomersall, S. R., Aitken, J. F., . . . Skinner, T. L. (2019). Peer support for the maintenance of physical activity and health in cancer survivors: the PEER trial - a study protocol of a randomised controlled trial. *BMC Cancer, 19*(1), 656. doi:10.1186/s12885-019-5853-4

Alfano, C. M., Peng, J., Andridge, R. R., Lindgren, M. E., Povoski, S. P., Lipari, A. M., . . . Janice, K. K. G. (2017). Inflammatory cytokines and comorbidity development in breast cancer survivors versus noncancer controls: Evidence for accelerated aging? *Journal of Clinical Oncology, 35*(2), 149-156. doi:10.1200/JCO.2016.67.1883

Arikawa, A. Y., Kaufman, B. C., Raatz, S. K., & Kurzer, M. S. (2018). Effects of a parallel-arm randomized controlled weight loss pilot study on biological and psychosocial parameters of overweight and obese breast cancer survivors. *Pilot and Feasibility Studies, 4*(1). doi:10.1186/s40814-017-0160-9

Augustin, L. S. A., Libra, M., Crispo, A., Grimaldi, M., De Laurentiis, M., Rinaldo, M., . . . et al. (2017). Low glycemic index diet, exercise and vitamin D to reduce breast cancer recurrence (DediCa): design of a clinical trial. *BMC cancer, 17*(1). doi:10.1186/s12885-017-3064-4

Azamian, A., Mobarekeh, B. G., Vismeh, Z., & Gohar, N. P. (2015). Effect of 12 weeks of selected pilates exercise training on serum adiponectin level and insulin resistance in female survivors of breast cancer and its role in prevention of recurrence. *Scientific Journal of Kurdistan University of Medical Sciences, 20*(5), 61-73.

Bahl, M., Ennis, M., Tannock, I. F., Hux, J. E., Pritchard, K. I., Koo, J., & Goodwin, P. J. (2005). Serum lipids and outcome of early-stage breast cancer: Results of a prospective cohort study. *Breast Cancer Research and Treatment, 94*(2), 135-144. doi:10.1007/s10549-005-6654-9

Befort, C. A., Klemp, J. R., Fabian, C., Perri, M. G., Sullivan, D. K., Schmitz, K. H., . . . Shireman, T. (2014). Protocol and recruitment results from a randomized controlled trial comparing group phone-based

versus newsletter interventions for weight loss maintenance among rural breast cancer survivors. *Contemporary Clinical Trials, 37*(2), 261-271. doi:10.1016/j.cct.2014.01.010

Bera, A., Russ, E., Srinivasan, M., Eidelman, O., Eklund, M., Hueman, M., . . . Srivastava, M. (2020a). Corrigendum to: Proteomic Analysis of Inflammatory Biomarkers Associated With Breast Cancer Recurrence. *Military medicine*. doi:10.1093/milmed/usaa082

Bera, A., Russ, E., Srinivasan, M., Eidelman, O., Eklund, M., Hueman, M., . . . Srivastava, M. (2020b). Proteomic Analysis of Inflammatory Biomarkers Associated With Breast Cancer Recurrence. *Military medicine, 185*(1), 669-675. doi:10.1093/milmed/usz254

Blackburn, G. L., & Wang, K. A. (2007). Dietary fat reduction and breast cancer outcome: results from the Women's Intervention Nutrition Study (WINS). *The American journal of clinical nutrition, 86*(3), s878-881.

Bobin-Dubigeon, C., Lefrançois, A., Classe, J. M., Joalland, M. P., & Bard, J. M. (2015). Paired measurement of serum amyloid A (SAA) and paraoxonase 1 (PON1) as useful markers in breast cancer recurrence. *Clin Biochem, 48*(16-17), 1181-1183. doi:10.1016/j.clinbiochem.2015.07.020

Bower, J. E., Greendale, G., Crosswell, A. D., Garet, D., Sternlieb, B., Ganz, P. A., . . . Cole, S. W. (2014). Yoga reduces inflammatory signaling in fatigued breast cancer survivors: a randomized controlled trial. *Psychoneuroendocrinology, 43*, 20-29. doi:10.1016/j.psyneuen.2014.01.019

Bramwell, V. H. C., Tuck, A. B., Chapman, J. A. W., Anborgh, P. H., Postenka, C. O., Al-Katib, W., . . . Chambers, A. F. (2014). Assessment of osteopontin in early breast cancer: Correlative study in a randomised clinical trial. *Breast Cancer Research, 16*(1). doi:10.1186/bcr3600

Calip, G. S., Malone, K. E., Gralow, J. R., Stergachis, A., Hubbard, R. A., & Boudreau, D. M. (2014). Metabolic syndrome and outcomes following early-stage breast cancer. *Breast Cancer Research and Treatment, 148*(2), 363-377. doi:10.1007/s10549-014-3157-6

Cormie, P., Singh, B., Hayes, S., Peake, J. M., Galvão, D. A., Taaffe, D. R., . . . Newton, R. U. (2016). Acute Inflammatory Response to Low-, Moderate-, and High-Load Resistance Exercise in Women With Breast Cancer-Related Lymphedema. *Integr Cancer Ther, 15*(3), 308-317. doi:10.1177/1534735415617283

Dittus, K. L., Harvey, J. R., Bunn, J. Y., Kokinda, N. D., Wilson, K. M., Priest, J., & Pratley, R. E. (2018). Impact of a behaviorally-based weight loss intervention on parameters of insulin resistance in breast cancer survivors. *BMC Cancer, 18*(1), 351. doi:10.1186/s12885-018-4272-2

Duggan, C., Irwin, M. L., Xiao, L., Henderson, K. D., Smith, A. W., Baumgartner, R. N., . . . McTiernan, A. (2011). Associations of insulin resistance and adiponectin with mortality in women with breast cancer. *Journal of Clinical Oncology, 29*(1), 32-39. doi:10.1200/JCO.2009.26.4473

Duggan, C., Wang, C. Y., Neuhouser, M. L., Xiao, L., Smith, A. W., Reding, K. W., . . . McTiernan, A. (2013). Associations of insulin-like growth factor and insulin-like growth factor binding protein-3 with mortality in women with breast cancer. *International Journal of Cancer, 132*(5), 1191-1200. doi:10.1002/ijc.27753

Fujii, T., Yajima, R., Takada, T., Sutoh, T., Morita, H., Yamaguchi, S., . . . Kuwano, H. (2014). Serum albumin and prealbumin do not predict recurrence in patients with breast cancer. *Anticancer Res, 34*(7), 3775-3779.

Furberg, A. S., Veierød, M. B., Wilsgaard, T., Bernstein, L., & Thune, I. (2004). Serum high-density lipoprotein cholesterol, metabolic profile, and breast cancer risk. *J Natl Cancer Inst, 96*(15), 1152-1160. doi:10.1093/jnci/djh216

Galanti, G., Stefani, L., & Gensini, G. (2013). Exercise as a prescription therapy for breast and colon cancer survivors. *Int J Gen Med, 6*, 245-251. doi:10.2147/ijgm.s42720

Gentry, A. L., Sereika, S. M., Casillo, F. E., Crisafio, M. E., Donahue, P. T., Grove, G. A., . . . Bender, C. M. (2018). Protocol for Exercise Program in Cancer and Cognition (EPICC): a randomized controlled trial of the

effects of aerobic exercise on cognitive function in postmenopausal women with breast cancer receiving aromatase inhibitor therapy. *Contemporary clinical trials*.

Ghosh, S., Hughes, D., Parma, D. L., Ramirez, A., & Li, R. (2014). Association of obesity and circulating adipose stromal cells among breast cancer survivors. *Molecular Biology Reports, 41*(5), 2907-2916. doi:10.1007/s11033-014-3146-1

Giganti, M. G., Tresoldi, I., Sorge, R., Melchiorri, G., Triossi, T., Masuelli, L., . . . Bei, R. (2016). Physical exercise modulates the level of serum MMP-2 and MMP-9 in patients with breast cancer. *Oncol Lett, 12*(3), 2119-2126. doi:10.3892/ol.2016.4887

Gnagnarella, P., Dragà, D., Baggi, F., Simoncini, M. C., Sabbatini, A., Mazzocco, K., . . . Maisonneuve, P. (2016). Promoting weight loss through diet and exercise in overweight or obese breast cancer survivors (InForma): Study protocol for a randomized controlled trial. *Trials, 17*(1). doi:10.1186/s13063-016-1487-x

Goodwin, P. J., Ennis, M., Pritchard, K. I., Trudeau, M. E., Koo, J., Madarnas, Y., . . . Hood, N. (2002). Fasting insulin and outcome in early-stage breast cancer: results of a prospective cohort study. *J Clin Oncol, 20*(1), 42-51. doi:10.1200/jco.2002.20.1.42

Hutnick, N. A., Williams, N. I., Kraemer, W. J., Orsega-Smith, E., Dixon, R. H., Bleznak, A. D., & Mastro, A. M. (2005). Exercise and lymphocyte activation following chemotherapy for breast cancer. *Med Sci Sports Exerc, 37*(11), 1827-1835. doi:10.1249/01.mss.0000175857.84936.1a

Irwin, M. L., Duggan, C., Wang, C. Y., Smith, A. W., McTiernan, A., Baumgartner, R. N., . . . Ballard-Barbash, R. (2011). Fasting C-peptide levels and death resulting from all causes and breast cancer: the health, eating, activity, and lifestyle study. *J Clin Oncol, 29*(1), 47-53. doi:10.1200/jco.2010.28.4752

Irwin, M. R., Olmstead, R., Breen, E. C., Witarama, T., Carrillo, C., Sadeghi, N., . . . et al. (2014). Tai chi, cellular inflammation, and transcriptome dynamics in breast cancer survivors with insomnia: a randomized controlled trial. *Journal of the national cancer institute. Monographs, 2014*(50), 295-301. doi:10.1093/jncimonographs/lgu028

Janelsins, M. C., Davis, P. G., Wideman, L., Katula, J. A., Sprod, L. K., Peppone, L. J., . . . Mustian, K. M. (2011). Effects of Tai Chi Chuan on insulin and cytokine levels in a randomized controlled pilot study on breast cancer survivors. *Clinical Breast Cancer, 11*(3), 161-170. doi:10.1016/j.clbc.2011.03.013

Johansen, J. S., Cintin, C., Jørgensen, M., Kamby, C., & Price, P. A. (1995). Serum YKL-40: a new potential marker of prognosis and location of metastases of patients with recurrent breast cancer. *Eur J Cancer, 31a*(9), 1437-1442. doi:10.1016/0959-8049(95)00196-p

Jones, L. W., Douglas, P. S., Eves, N. D., Marcom, P. K., Kraus, W. E., Herndon Ii, J. E., . . . Peppercorn, J. (2010). Rationale and design of the Exercise Intensity Trial (EXCITE): A randomized trial comparing the effects of moderate versus moderate to high-intensity aerobic training in women with operable breast cancer. *BMC Cancer, 10*. doi:10.1186/1471-2407-10-531

Knobf, M. T., & Coviello, J. (2011). Lifestyle interventions for cardiovascular risk reduction in women with breast cancer. *Current Cardiology Reviews, 7*(4), 250-257. doi:10.2174/157340311799960627

Kus, T., Cinkir, H. Y., Aktas, G., & Abali, H. (2019). Hepatosteatosis may predict late recurrence of breast cancer: A single-center observational study. *Current Problems in Cancer, 43*(6). doi:10.1016/j.currproblcancer.2019.01.002

Larkey, L., Huberty, J., Pedersen, M., & Weihs, K. (2016). Qigong/Tai Chi Easy for fatigue in breast cancer survivors: Rationale and design of a randomized clinical trial. *Contemp Clin Trials, 50*, 222-228. doi:10.1016/j.cct.2016.08.002

Long Parma, D., Hughes, D. C., Ghosh, S., Li, R., Treviño-Whitaker, R. A., Ogden, S. M., & Ramirez, A. G. (2015). Effects of six months of Yoga on inflammatory serum markers prognostic of recurrence risk in breast cancer survivors. *Springerplus, 4*, 143. doi:10.1186/s40064-015-0912-z

McEligot, A. J., Rock, C. L., Flatt, S. W., Newman, V., Faerber, S., & Pierce, J. P. (1999). Plasma carotenoids are biomarkers of long-term high vegetable intake in women with breast cancer. *Journal of Nutrition, 129*(12), 2258-2263.

Meyerhardt, J. A., Irwin, M. L., Jones, L. W., Zhang, S., Campbell, N., Brown, J. C., . . . et al. (2020). Randomized phase II trial of exercise, metformin, or both on metabolic biomarkers in colorectal and breast cancer survivors. *JNCI cancer spectrum, 4*(1). doi:10.1093/jncics/pkz096

Nelson, S. H., Brasky, T. M., Patterson, R. E., Laughlin, G. A., Kritz-Silverstein, D., Edwards, B. J., . . . LaCroix, A. Z. (2017). The association of the c-reactive protein inflammatory biomarker with breast cancer incidence and mortality in the women's health initiative. *Cancer Epidemiology Biomarkers and Prevention, 26*(7), 1100-1106. doi:10.1158/1055-9965.EPI-16-1005

Nock, N. L., Owusu, C., Kullman, E. L., Austin, K., Roth, B., Cerne, S., . . . Berger, N. A. (2013). A Community-Based Exercise and Support Group Program in African-American Breast Cancer Survivors (ABCs). *J Phys Ther Health Promot, 1*(1), 15-24. doi:10.18005/pthp0101003

Nuri, R., Mahmudieh, B., Damirchi, A., Rahmani-Nia, R., Rahnama, N., & Emami, H. (2014). Changes in IGF axis and some binding proteins in postmenopausal women with breast cancer after 15 weeks of combined exercise training. *Journal of Zanjan University of Medical Sciences and Health Services, 22*(91), 1-10.

Oh, S. W., Park, C., Lee, E. S., Yoon, Y. S., Lee, E. S., Park, S. S., . . . Ro, J. (2011). Adipokines, insulin resistance, metabolic syndrome, and breast cancer recurrence: A cohort study. *Breast Cancer Research, 13*(2). doi:10.1186/bcr2856

Owusu, C., Nock, N. L., Hergenroeder, P., Austin, K., Bennet, E., Cerne, S., . . . Berger, N. A. (2020). IMPROVE, a community-based exercise intervention versus support group to improve functional and health outcomes among older African American and non-Hispanic White breast cancer survivors from diverse socioeconomic backgrounds: Rationale, design and methods. *Contemporary Clinical Trials, 92*. doi:10.1016/j.cct.2020.106001

Pierce, B. L., Ballard-Barbash, R., Bernstein, L., Baumgartner, R. N., Neuhouser, M. L., Wener, M. H., . . . Ulrich, C. M. (2009). Elevated biomarkers of inflammation are associated with reduced survival among breast cancer patients. *Journal of Clinical Oncology, 27*(21), 3437-3444. doi:10.1200/JCO.2008.18.9068

Pudkasam, S., Pitcher, M., Fisher, M., O'Connor, A., Chinlumprasert, N., Stojanovska, L., . . . Apostolopoulos, V. (2020). The PAPHIO study protocol: a randomised controlled trial with a 2 x 2 crossover design of physical activity adherence, psychological health and immunological outcomes in breast cancer survivors. *BMC Public Health, 20*(1), 696. doi:10.1186/s12889-020-08827-x

Ramirez, A. G., Parma, D. L., Muñoz, E., Mendoza, K. D., Harb, C., Holden, A. E. C., & Wargovich, M. (2017). An anti-inflammatory dietary intervention to reduce breast cancer recurrence risk: Study design and baseline data. *Contemporary Clinical Trials, 57*, 1-7. doi:10.1016/j.cct.2017.03.009

Reeves, M. M., Terranova, C. O., Erickson, J. M., Job, J. R., Brookes, D. S. K., McCarthy, N., . . . et al. (2016). Living well after breast cancer randomized controlled trial protocol: evaluating a telephone-delivered weight loss intervention versus usual care in women following treatment for breast cancer. *BMC cancer, 16*(1). doi:10.1186/s12885-016-2858-0

Repka, C. P., & Hayward, R. (2018). Effects of an Exercise Intervention on Cancer-Related Fatigue and Its Relationship to Markers of Oxidative Stress. *Integrative Cancer Therapies, 17*(2), 503-510. doi:10.1177/1534735418766402

Rhone, P., Bielawski, K., Ziołkowska, K., Rość, D., & Ruszkowska-Ciastek, B. (2019). Low pre-treatment count of circulating endothelial progenitors as a prognostic biomarker of the high risk of breast cancer recurrence. *Journal of Clinical Medicine, 8*(11). doi:10.3390/jcm8111984

Rock, C. L., Pande, C., Flatt, S. W., Ying, C., Pakiz, B., Parker, B. A., . . . Nichols, J. F. (2013). Favorable changes in serum estrogens and other biologic factors after weight loss in breast cancer survivors who are overweight or obese. *Clinical Breast Cancer, 13*(3), 188-195. doi:10.1016/j.clbc.2012.12.002

Rothammer, A., Sage, E. K., Werner, C., Combs, S. E., & Multhoff, G. (2019). Increased heat shock protein 70 (Hsp70) serum levels and low NK cell counts after radiotherapy - potential markers for predicting breast cancer recurrence? *Radiat Oncol, 14*(1), 78. doi:10.1186/s13014-019-1286-0

Sestak, I., Dowsett, M., Ferree, S., Baehner, F. L., & Cuzick, J. (2016). Retrospective analysis of molecular scores for the prediction of distant recurrence according to baseline risk factors. *Breast Cancer Res Treat, 159*(1), 71-78. doi:10.1007/s10549-016-3868-y

Skouroliakou, M., Grosomanidis, D., Massara, P., Kostara, C., Papandreou, P., Ntountaniotis, D., & Xepapadakis, G. (2018). Serum antioxidant capacity, biochemical profile and body composition of breast cancer survivors in a randomized Mediterranean dietary intervention study. *European Journal of Nutrition, 57*(6), 2133-2145. doi:10.1007/s00394-017-1489-9

Sprod, L. K., Janelsins, M. C., Palesh, O. G., Carroll, J. K., Heckler, C. E., Peppone, L. J., . . . Mustian, K. M. (2012). Health-related quality of life and biomarkers in breast cancer survivors participating in tai chi chuan. *Journal of Cancer Survivorship, 6*(2), 146-154. doi:10.1007/s11764-011-0205-7

Stoll, B. A. (1996). Diet and exercise regimens to improve breast carcinoma prognosis. *Cancer, 78*(12), 2465-2470. doi:10.1002/(SICI)1097-0142(19961215)78:12<2465::AID-CNCR3>3.0.CO;2-J

Stolley, M., Sheean, P., Gerber, B., Arroyo, C., Schiffer, L., Banerjee, A., . . . Sharp, L. (2017). Efficacy of a weight loss intervention for african American breast cancer survivors. *Journal of Clinical Oncology, 35*(24), 2820-2828. doi:10.1200/JCO.2016.71.9856

Študentová, H., Vitásková, D., Šrámek, V., Indráková, J., Adam, T., Juráňová, J., . . . Melichar, B. (2015). Correlations of neutrophil-to-lymphocyte, lymphocyte-to-monocyte and platelet-to-lymphocyte ratios with biomarkers of atherosclerosis risk and inflammatory response in patients with a history of breast cancer. *Pteridines, 26*(4), 161-172. doi:10.1515/pterid-2015-0009

Thomson, C. A., Stopeck, A. T., Bea, J. W., Cussler, E., Nardi, E., Frey, G., & Thompson, P. A. (2010). Changes in body weight and metabolic indexes in overweight breast cancer survivors enrolled in a randomized trial of low-fat vs. reduced carbohydrate diets. *Nutrition and Cancer, 62*(8), 1142-1152. doi:10.1080/01635581.2010.513803

Walter, K. R., Ford, M. E., Gregoski, M. J., Kramer, R. M., Knight, K. D., Spruill, L., . . . Turner, D. P. (2019). Advanced glycation end products are elevated in estrogen receptor-positive breast cancer patients, alter response to therapy, and can be targeted by lifestyle intervention. *Breast Cancer Res Treat, 173*(3), 559-571. doi:10.1007/s10549-018-4992-7

Zhang, J. Y., Liao, Y. H., Lin, Y., Liu, Q., Xie, X. M., Tang, L. Y., & Ren, Z. F. (2019). Effects of tea consumption and the interactions with lipids on breast cancer survival. *Breast Cancer Res Treat, 176*(3), 679-686. doi:10.1007/s10549-019-05253-5
